# Revitalizing the US Youth Presidential Fitness Test: Are States Prepared to Support Implementation?

**DOI:** 10.5888/pcd23.250456

**Published:** 2026-04-30

**Authors:** Chad M. Killian, Emily M. D’Agostino, Ben D. Kern, Christina Y. Kim, Devon King, Shannan E. Albeke, Hannah R. Thompson

**Affiliations:** 1 Department of Kinesiology, College of Health and Human Services, University of New Hampshire, Durham, New Hampshire; 2 Department of Orthopaedic Surgery, Duke University School of Medicine, Durham, North Carolina; 3 Department of Population Health Sciences, Duke University School of Medicine, Durham, North Carolina; 4 Duke Clinical Research Institute, Duke University School of Medicine, Durham, North Carolina; 5 Division of Kinesiology and Health, College of Health Sciences, University of Wyoming, Laramie, Wyoming; 6 Department of Biostatistics and Bioinformatics, Duke University School of Medicine, Durham, North Carolina; 7 Wyoming Geographic Information Science Center, School of Computing, University of Wyoming, Laramie, Wyoming; 8 Nutrition Policy Institute, University of California Agriculture and Natural Resources, Oakland, California; 9 Community Health Sciences, School of Public Health, University of California Berkeley, Berkeley, California

## Abstract

Our objective was to describe policy infrastructure of elementary school fitness testing at the state level and to assess states’ readiness for implementation of the recently reinstated Presidential Fitness Test. We conducted a cross-sectional policy analysis by using data from the State of the States Policy Report and used the Classification of Laws Associated with School Students to create a standardized readiness index. Only 5 states required recommended weekly minutes of physical education, 24 had no fitness testing requirements, 6 mandated testing results reporting, and most delegated compliance monitoring locally. State policies are not positioned to support large-scale standardized fitness testing, indicating a need for coordinated reform.

**Summary**

**What is already known on this topic?**

Youth fitness testing provides reliable, valid indicators of child health to support population-level public health surveillance and chronic disease prevention. State policy plays a role in provision of physical education and fitness testing implementation in K–12 schools.

**What is added by this report?**

We conducted the first state-by-state assessment of policy readiness of elementary school fitness testing and found substantial gaps in policy infrastructure.

**What are the implications for public health practice?**

Weak and inconsistent state policy infrastructures limit feasibility of implementing standardized, large-scale fitness testing, indicating the need for coordinated policy reform.

## Objective

In July 2025, President Trump amended Executive Order (EO) 13265 to “establish the President’s Council on Sports, Fitness, and Nutrition, and to reestablish the Presidential Fitness Test,” signaling renewed attention to youth physical activity and establishing it as a national priority (1). The order did not include mandates, timelines, or enforcement mechanisms for implementation, which leaves adoption contingent on existing state policy infrastructure.

School-based fitness testing, such as the Presidential Fitness Test (PFT) and FitnessGram, involves standardized health-related field assessments of students’ aerobic capacity, muscular strength and endurance, flexibility, and body composition. Standardized, high-quality (ie, psychometrically sound and criterion-based) school-based fitness testing has potential to provide wide-ranging benefits (2). For example, youth fitness testing surveillance offers public health officials timely population-level data to support identifying fitness patterns and mitigating disparities in fitness to reduce morbidity and mortality rates (3). Specifically, school-based fitness testing can guide physical activity programming and physical education (PE) interventions and provide relevant data for clinical practice. To this end, the Institute of Medicine and the National Academies of Sciences, Engineering, and Medicine have called for the expansion of school-based physical fitness testing in support of population-level health (4).

State policy shapes PE opportunities and implementation. Because fitness testing is typically administered during PE classes by PE teachers, state policies that govern PE instructional time, staffing, data collection, and reporting constitute essential infrastructure for regular and consistent implementation (2). For a nationwide fitness testing program like the PFT to be successful, it must be implemented within a well-supported and executed PE infrastructure backed by policies that promote adequate PE instructional time, a trained PE teacher workforce, consistent standardized fitness testing data collection and reporting, and mechanisms for accountability and enforcement (5). Since elementary schools represent the earliest and most universal points of student access, adequate PE infrastructure at this level is critical for scaled implementation. However, this structural environment is generally lacking (6,7), and prior efforts to generate national estimates of youth physical fitness have been limited due to insufficient and fragmented data (3). Therefore, the purpose of this article is to describe current state-level elementary PE and fitness testing policy infrastructure in the US and assess readiness to implement high-quality fitness testing at scale in our nation’s schools.

## Methods

We conducted a cross-sectional policy analysis of all 50 US states by using data from the State of the States Policy Report (8). This report, which is current through December 2024, synthesized codified, publicly available state policies identified through systematic legal searches of statutes, administrative codes, regulations, and legislative acts by using Fastcase (Fastcase Inc) and state education department repositories. Nonbinding guidance and local materials were excluded. Two researchers independently coded elementary-level PE policy indicators (n = 8), which were selected a priori on the basis of their relevance to fitness testing implementation. Indicators were related to 1) PE instructional time, teacher qualification, and standards; 2) health-related fitness testing requirements, assessment instruments, and reporting mandates; and 3) PE-specific policy oversight and enforcement mechanisms. Coding adhered to the most recent Classification of Laws Associated with School Students (CLASS) protocols and variable definitions (9) where applicable and was supplemented by custom variables to capture policy distinctions not reflected by CLASS. Each variable was scored by using a construct-specific ordinal scale, with higher values indicating stronger and more specific policy requirements. Scale ranges varied by indicator on an ordinal scale (eg, 0–1 to 0–5) (Table). Discrepancies between coders were reconciled through group discussion and consensus. Scores were then standardized (range, 0–1) and averaged to create a composite fitness testing policy readiness index. Each raw score was divided by its maximum possible value to yield a proportional score that reflected the degree to which each state met the strongest policy criteria for that variable in support of composite fitness testing policy readiness:

**Table:** Distribution of State Scores, by Policy Variable, Analysis of Elementary Physical Education and Fitness Testing Policy Infrastructure, United States, 2024

Policy assessment variable (score **a**; data scoring criteria)	N (%)
**Fitness assessment strength (score = 0–4; CLASS)**	
No requirement or recommendation (score = 0)	24 (48)
State only recommends assessment (score = 1)	11 (22)
State requires districts to assess at least once, components unspecified (score = 2)	7 (14)
State requires assessment >1 time, specifying cardiovascular endurance, muscular strength, muscular endurance, flexibility, and body composition (score = 3)	5 (10)
State requires annual assessments specifying cardiovascular endurance, muscular strength, muscular endurance, flexibility, and body composition (score = 4)	3 (6)
**Fitness assessment reporting (score = 0–3; custom)**	
No requirement or recommendation (score = 0)	36 (72)
State assigns local monitoring and/or reporting (eg, parents, school boards) (score = 1)	4 (8)
State requires submission of fitness reports to a state agency (score = 2)	6 (12)
State requires public reporting (eg, dashboards, report cards) (score = 3)	4 (8)
**Fitness assessment type (score = 0–4; custom)**	
No requirement or recommendation (score = 0)	17 (34)
State only recommends assessments without component specification (score = 1)	17 (34)
State requires use of any assessment with specified components (eg, cardiovascular endurance, muscular strength, muscular endurance, flexibility, or body composition) (score = 2)	7 (14)
State requires use of a state-developed or state-selected standardized assessment (score = 3)	5 (10)
State requires use of a standardized national assessment (ie, FitnessGram, Brockport Physical Fitness Test) (score = 4)	4 (8)
**Fitness standards (score = 0–3; custom)**	
No mention of fitness (score = 0)	0 (0)
Fitness implied conceptually (eg, “health” or “wellness” goals) but not named explicitly (score = 1)	0 (0)
Fitness mentioned generally but without specified components or measurable benchmarks (score = 2)	0 (0)
Fitness explicitly mentioned with specific components (eg, cardiovascular endurance, muscular strength, flexibility, or body composition) (score = 3)	50 (100)
**Fitness enforcement level (score = 0–4; custom)**	
No PE or PA requirement (score = 0)	1 (2)
State recommends local oversight of PE or PA compliance (score = 1)	0 (0)
State assigns local oversight of PE or PA compliance (ie, districts must adopt policies, monitor compliance, or submit reports) (score = 2)	33 (66)
State assigns state oversight of PE or PA without enforcement consequences (eg, required reporting but no compliance penalties) (score = 3)	11 (22)
State requires direct state oversight of PE or PA with compliance penalties (ie, loss of state aid, accreditation penalties, or corrective action orders) (score = 4)	5 (10)
**Indirect oversight (score = 0–1; custom)**	
No indication (score = 0)	9 (18)
Monitoring conducted by state for PE or PA indirectly (eg, through accreditation, school improvement, wellness plans, or general standards compliance processes) (score = 1)	41 (82)
**PE teacher licensure requirement (score = 0–3; CLASS)**	
No requirement (score = 0)	1 (2)
State recommends PE teacher licensure (score = 1)	1 (2)
State requires licensure for newly hired PE teachers (score = 2)	0 (0)
State requires licensure for all PE teachers (score = 3)	48 (96)
**Required PE time (min/week)^b^ (score = 0–5; CLASS)**	
No requirement (score = 0)	5 (10)
State recommends a PE time requirement (score = 1)	6 (12)
State requires PE for up to 60 min/week (score = 2)	26 (52)
State requires PE for 60 to <90 min/week (or the equivalent in credit[s] based on the Carnegie unit^c^) (score = 3)	2 (4)
State requires PE for 90 to <150 min/week (or the equivalent in credit[s] based on the Carnegie unit^c^) (score = 4)	6 (12)
State requires PE for ≥150 min/per week (or the equivalent in credit[s] based on the Carnegie unit^c^) (score = 5)	5 (10)

Abbreviations: CLASS, Classification of Laws Associated with School Students; PA, physical activity; PE, physical education.

a Each variable was scored on an ordinal scale (range, 0–1 to 0–5) that reflected the strength and specificity of policy requirements.

b Refers to public school districts.

c A Carnegie unit is a time-based academic credit standard used in US education that is awarded for completion of a course that meets for approximately 1 class period per day over an academic year and totals approximately 120 instructional hours.

The proportional score*_i_* is equal to the raw score*_i_* divided by the maximum possible score*_i_*, where (R_i) = raw score for state (i) and (M_i) = maximum possible value for that variable, which results in a proportional score of *P_i_* = *R_i_/M_i_.*

This approach ensured that all variables contributed equally to the composite score, regardless of their original scoring range. Frequencies and proportions were calculated to summarize state-level distributions for each policy indicator, and composite readiness scores were summarized by using means and ranges.

## Results

The mean readiness score for composite state-level fitness testing for US elementary schools was 0.57 (range, 0.36–0.75), which indicates moderate but inconsistent policy infrastructure nationwide to support standardized, high-quality elementary school fitness testing. All states explicitly identified health-related fitness within PE standards or curriculum framework, and nearly all (n = 48 [96%]) required PE licensed instructors to teach elementary PE (Figure, Table). However, only 5 states (10%) mandated the US Department of Health and Human Services recommended 150 minutes per week of elementary PE time, and most (n = 37 [74%]) required zero to less than 60 weekly minutes. Twenty-four states (48%) had no requirements or recommendations for fitness testing, while 11 (22%) recommended fitness testing and only 3 required it annually. A total of 6 states (12%) mandated submission of fitness testing results to state agencies, and only 4 (8%) required that results be made publicly accessible. Most states (n = 33 [66%]) assigned local control to monitor PE and fitness testing compliance. Only 16 states (32%) required state oversight, but most of these (n = 11 [22%]) lacked any enforcement consequences. 

**Figure:**
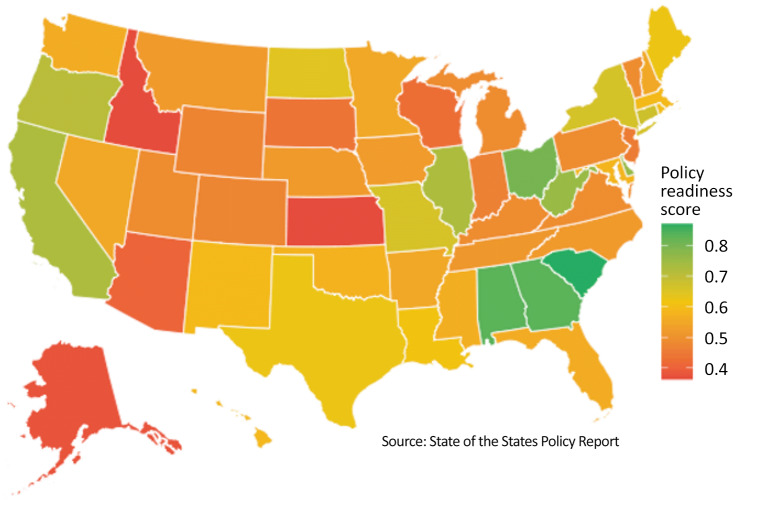
US composite scores of states’ fitness testing policy readiness for US elementary schools, drawn from State of the States Policy Report data. Scores were standardized (range, 0–1) and averaged to create a composite fitness testing policy readiness index. Higher scores indicate stronger physical education policies. Source: King D, et al (8).

## Discussion

Foundational state-level policies necessary to support scaled elementary school-based fitness testing are inconsistent across the US, and state policy infrastructure and readiness is generally low. Taken together, these findings show that states are not currently positioned to support standardized, large-scale fitness testing outlined under the reestablished PFT. Although most states (96%) maintained appropriate PE workforce licensing requirements and promoted appropriate learning standards and curricular foundations for teaching fitness in PE, limitations in elementary PE time mandates continue to result in PE access disparities (6,7,10). Furthermore, absence of coordinated policies for conducting, reporting, and enforcing consistent high-quality school-based fitness testing is problematic (3,8). Weak or absent reporting mandates also limit integration of fitness testing data in to broader public health data systems, which reduces possibilities to support population-level health promotion and surveillance. For comparison, data from the most recent (2016) Shape of the Nation report identified only 16 (32%) states mandating school-based fitness testing and 10 (20%) reporting data publicly (11), which indicates limited policy progress over the last decade with differences likely reflecting updated policy (8,11). Strengthening state-level policies that build school capacity for consistent and appropriate fitness testing and promote school reporting and integration of data within public health data systems would improve national surveillance and allow population-level fitness data to inform chronic disease prevention and intervention programs, health-equity monitoring, PE practice and policy evaluation, and data-informed resource allocation (3).

Advancing national school-based fitness testing requires state policy reform to increase access to PE and fitness testing, along with reporting mandates, to ensure high-quality and sustainable implementation. For the PFT to provide a meaningful lever for youth public health promotion and surveillance, systematic state policy reform and resulting school-level PE infrastructure changes are necessary.

To our knowledge, our study presents the first analysis that reports state policy readiness to implement high-quality fitness testing at scale in our nation’s elementary schools. Although it draws from the most recent (2024) policy infrastructure data (8), it was limited to active state-level legislation and did not capture district-level or agency-level policies. Some regional patterns were also observed; however, this descriptive study was not designed to explain determinants of state differences. Future studies integrating policy, implementation, and contextual data are needed to further understand variations in readiness.
